# The Impact on Staff of Working with Personality Disordered Offenders: A Systematic Review

**DOI:** 10.1371/journal.pone.0136378

**Published:** 2015-08-25

**Authors:** Mark C. Freestone, Kim Wilson, Rose Jones, Chris Mikton, Sophia Milsom, Ketan Sonigra, Celia Taylor, Colin Campbell

**Affiliations:** 1 East London NHS Foundation Trust, London, United Kingdom; 2 Queen Mary University of London, London, United Kingdom; 3 Northumberland, Tyne and Wear NHS Trust, Newcastle, United Kingdom; 4 World Health Organisation, Geneva, Switzerland; 5 South London and the Maudsley NHS Trust, London, United Kingdom; 6 King’s College London, London, United Kingdom; Carl von Ossietzky University of Oldenburg, GERMANY

## Abstract

**Background:**

Personality disordered offenders (PDOs) are generally considered difficult to manage and to have a negative impact on staff working with them.

**Aims:**

This study aimed to provide an overview of studies examining the impact on staff of working with PDOs, identify impact areas associated with working with PDOs, identify gaps in existing research,and direct future research efforts.

**Methods:**

The authors conducted a systematic review of the English-language literature from 1964–2014 across 20 databases in the medical and social sciences.

**Results:**

27 papers were included in the review. Studies identified negative impacts upon staff including: negative attitudes, burnout, stress, negative counter-transferential experiences; two studies found positive impacts of job excitement and satisfaction, and the evidence related to perceived risk of violence from PDOs was equivocal. Studies demonstrated considerable heterogeneity and meta-analysis was not possible. The overall level of identified evidence was low: 23 studies (85%) were descriptive only, and only one adequately powered cohort study was found.

**Conclusions:**

The review identified a significant amount of descriptive literature, but only one cohort study and no trials or previous systematic reviews of literatures. Clinicians and managers working with PDOs should be aware of the potential impacts identified, but there is an urgent need for further research focusing on the robust evaluation of interventions to minimise harm to staff working with offenders who suffer from personality disorder.

## Introduction

People with personality disorder (PD) are generally considered particularly difficult to manage, treat, and interact with; they are often disliked by mental health professionals [1]; and are widely believed to have a negative impact on staff working with them [2]. Previous studies have shown that staff competency, and investment in staff training, are associated with reduced staff turnover and improved service and treatment outcomes [3, 4].

Forensic services for offenders who have a personality disorder (Personality disordered offenders: PDOs) face the additional difficulty of working with individuals who have had previous contact with criminal justice services and may have histories of serious violent and/or sexually deviant behaviour. Although necessary, having confident and well-supported staff may still not be sufficient for ensuring the effectiveness of PD treatments in forensic services.

It has been claimed that the impact on staff of working in a forensic PD service was an important contributory factor in treatment failures and eventual closures of such units in the past, e.g. at Ashworth Hospital as described in the Blom-Cooper and Fallon Reports [5, 6] and elsewhere [7]. It was posited that extended contact with PDOs can be challenging, possibly traumatic, and requires significant specialised training and/or additional supervision to be managed effectively. This could be due to increased risk of interpersonal violence or aggression (for example, a higher number of aggressive or ‘untoward’ incidents in services for PDOs), or psychosocial mechanisms such as manipulation, splitting or ‘burnout’ due to extended periods of emotionally draining interactions.

Existing research directly examining the impact on staff of working with PDOs [8, 9] and studies on the impact of psychopaths on clinicians [10–14] suggest that working with this population can have a detrimental effect on staff. In addition, there is vast literature that is relevant to differing degrees to this topic, such as that relating to the impact of working with prisoners on prison staff or the impact on staff of working in forensic, general adult or other psychiatric services. This literature shows that to intervene to reduce the negative impact PDOs have on staff, it is necessary to a) identify the exact nature and intensity of the impact, i.e. the outcomes, and b) develop empirically supported models of the causes, moderators, and mediators of the impact.

The literature relevant to the impact on staff of working with PDOs has, to date, not been systematically reviewed.

## Objectives

This study had four key objectives:

1. To provide an overview of existing studies examining the impact on staff of working in treatment services for PDOs.
2. To identify the core impact areas (positive or negative) associated with working with PDOs.
3. To identify gaps in existing research on this topic.
4. To direct future research efforts.

## Methods

### Study inclusion criteria

A systematic review was carried out of studies that address the research question directly, i.e. the impact, whether physical, psychological or behavioural, on staff working in treatment services with PDOs. The study considered English language studies only and focused on any setting (inpatient and community) in which healthcare or social care professionals (nurses, doctors, psychologists, prison officers, social workers, etc.) were in contact with PDOs.

The aim of the study was to perform a systematic review of the available evidence, which did not initially exclude studies on grounds of study design alone. This implied the use of a range of different critical appraisal tools and approaches to synthesizing what would probably be heterogeneous evidence, including the use of diagrams summarizing the range, quality, and type of research evidence available [15].

From the early design stages, it was anticipated that this would be a review of complex evidence, not of a single clearly defined intervention/treatment. This implies that the review itself would:

- Be relatively complex, extensive, and time-consuming, because of the need to review very heterogeneous types of evidence.
- Consider a wide range of questions, the inclusion criteria for the studies would be complex, and would have to take the findings of a number of different—both qualitative and quantitative—study designs into account.
- Involve a complex search for studies including a review of the grey literature.
- Require a range of approaches to quality assessment, and would not focus just on outcomes, but processes.

Studies were selected according to a Population, Exposure and Outcome (PEO) algorithm described below. No specifications were made regarding outcome and both negative and positive variance of outcome measures were included as search terms.

### Search methods for identification of studies

#### Electronic Searches

Keywords, abstract and title (ab.ti) were searched in the following electronic databases, using the search terms detailed in Table 1. The search was conducted in January 2014 and restricted to articles published since 1963:

- CINAHL
- Criminal Justice Abstracts
- ASSIA
- Social Care Online
- OVID—MEDLINE
- OVID—British Nursing Index
- OVID—EMBASE
- OVID—PsycINFO
- OVID—HMIC
- HSTAT
- NCJRS Abstracts
- HSRProj
- Regard
- Home Office Research
- Social Services Abstracts
- Social Science Citation Index
- CRISP
- CRD

**Table 1:** Search terms used for systematic review.

Population	mental health worker or mental health staff or psychiatrist or doctor or physician or personnel or employee or psychologist or nurs$3 or social worker or therapist or psychotherapist or analyst or psychoanalyst or counsellor or clinician or staff
Exposure	personality disorder or psychopath$2 or antisocial or borderline or narcissistic or axis II or personality pathology or characterological or characterAND forensic or secure or special hospital or prison treatment or prison hospital or therapeutic community or Grendon or offender
Outcome	impact or reaction or outcome or countertransferen$4 or emotion or experience or response or effect or stress or strain or burnout or attitude or perception or manipulation or job satisfaction or job dissatisfaction or mental health or well-being or anxiety or violen$2 or assault$5 or attack depression or symptom or psychosomatic or health or physiolog$4 or drug or alcohol$3 or substance or commitment or involvement or frustration or sick day or absenteeism or performance or turnover or overload or suicid$2 or withdrawal or organizational citizenship or general health questionnaire

The Campbell and Cochrane Collaboration databases of systematic reviews were also searched for pre-existing systematic reviews on similar topics.

#### Hand Searches

The top five journals (see S1 Appendix) containing the highest number of eligible studies were hand-searched for further relevant papers in relation to a 10 year time period; additionally, the 10 authors who featured most in cited literature were contacted as 'expert commentators' and asked to identify any 'grey' literature that may be in existence. Studies identified by either route were then reviewed for inclusion.

### Data collection and analysis

#### Selection of articles

To select articles on the basis of relevance, we employed a PEO algorithm, which was applied as follows and operationalised for electronic searches in Table 1:

- Population: Any individual or group of individuals working professionally with offenders or mental health patients.
- Exposure: The population must have been exposed to individuals diagnosed with either a personality disorder or a psychopathic disorder during the course of their daily work. Those individuals must also have committed a crime or be classified as ‘forensic’ patients. The setting must be one where individuals are detained for reasons of offending or socially unacceptable behaviour: forensic inpatient wards or prisons.
- Outcome: Any outcome relating to staff wellbeing, physical or mental health.

The titles and abstracts of all potentially suitable studies were inspected by review authors (CC, MF, KS, SM or KW) independently. The full text of articles meeting the inclusion criteria were retrieved and reviewed independently by one author. Where it was unclear whether or not the criteria had been met, articles were re-reviewed by MF for final inclusion or exclusion.

#### Data extraction and management

Data from each article were extracted independently by two of five authors (CC, MF, KS, SM or KW), and then the extraction documents compared to check consistency of data extraction. Any disagreement was discussed with an additional author and, where necessary, the author(s) of the study were contacted for further information.

Studies were expected to feature a wide range of methodologies, including qualitative and single-case studies, which prompted a detailed consideration of data extraction methods to ensure some degree of comparability. Due to the expectation of a high degree of study heterogeneity, different extraction tables were used dependent on the methodology employed (see S2 Appendix).

- Single studies employing quantitative data
  These studies were assessed for i) construct validity of outcome measures or concepts used; ii) validity of statistical conclusion (based on sample size, effect size and power calculations if available); iii) internal validity (coherence of argument); iv) external validity (applicability outside the given setting and congruence with other literature); and v) descriptive validity (comprehensiveness of reporting; description of outcomes).
- Single studies employing qualitative data
  Qualitative studies were evaluated in terms of a range of factors including: relevance, clarity of research question, appropriateness of design to question, context, sampling, data collection and analysis, audit trail, reflexivity, triangulation, respondent validation, and attention to negative cases.
- Expert opinion papers
  Clinical vignettes, single-case studies and editorials were expected to be highly heterogeneous and data were extracted in the form of a general summary of these papers and constructs or topics of interest to the review, rather than a systematic account of study quality.

As part of the data extraction process, studies were graded according to the level of evidence they represented, based on the levels of evidence identified by the Oxford Centre for Evidence-Based Medicine [16]. Each study was graded on the ‘evidence of harms’ scale ranging from Level 1 (Systematic Review of Trials) to Level 5 (Mechanism-based reasoning, such as an clinical piece). Consistent with the CEBM guidelines, studies reaching a certain level (e.g. Level 3:Non-randomised cohort) were downgraded (e.g. to Level 4: Case control) if they were inadequately powered or imprecise in their reporting. Purely descriptive studies, including cross-sectional studies qualitative evidence, were graded as Level 5 evidence.

#### Data synthesis

Due to the likely high level of study heterogeneity, data were summarised in narrative and tabular formats:

- Tables summarizing the main features of each study;
- Narrative analysis of each study, summarised by a paragraph about each study including information of sample size, design, setting, location and main effects;
- Cross-study synthesis (description of amount of information found; overall statement of the effect of exposure; summary of the results of individual studies).

The results were not subjected to naturalistic meta-analysis due to the small number of robust quantitative studies with comparable outcomes identified.

## Results

### Description of included studies

#### Summary of search results

**Fig 1:**
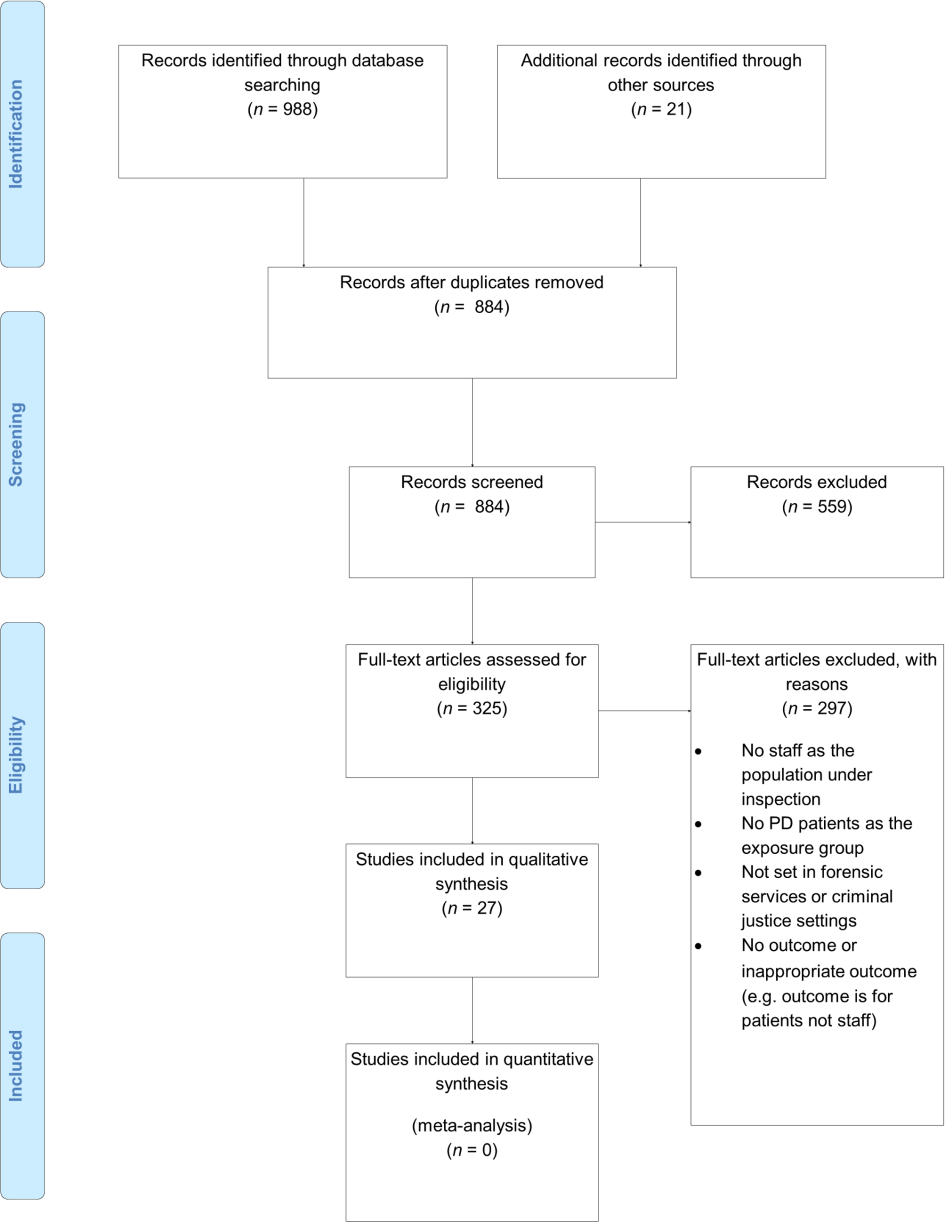
PRISMA Flow chart for selection of studies included in the systematic review.

Initial searches of electronic databases generated 988 possible articles, which were then reviewed for relevance. Eventually 27 articles were deemed of sufficient relevance and selected for data extraction (Fig 1). A full summary list of these studies is given in Table 2.

**Table 2:** Summary of all identified studies.

Ref.	Name	Achieved sample	Methodology	Evidence Grade	Outcome: Identified Themes and Findings	Relevance
18	Mason et al. (2010a)	545 staff members: as Mason (2010b) plus 129 staff from other professional groups (3 from high, 87 from medium, 39 from low secure)	Quantitative, C/S: Survey employing a questionnaire about clinical outcomes for patient groups	5	For both nursing and other professional groups, patients with a mental illness were considered more treatable, more responsive to clinical intervention and less of a management concern than PD patients.	High
19	Mason et al. (2010b)	416 Forensic Psychiatric nurses: 122 from high secure; 159 from medium secure; 135 from low secure settings	Quantitative, C/S: Survey employing a questionnaire about clinical outcomes for patient groups	5	PD diagnosis more of a management concern compared to MI diagnosis, which was considered more clinically treatable. Focus on the management of PDOs across all three security areas, implying nurses consider PDOs difficult to treat or engage in treatment and lack confidence in the outcome or efficacy of clinical interventions for this group. Caring for PDOs in high secure may impact on perceptions of whether a positive clinical outcome is achievable.	High
26	Bowers et al. (2006)	73 Prison officers from UK DSPD Prison Unit; 59 at 8 month follow up 37 at 16 month follow up	Quantitative, Longitudinal: Survey design involving administration of the Attitudes to Personality Disorder Questionnaire	3	Over time staff attitudes to PDOs became more negative, but not significantly (*F*(2,35) = 2.67, *p* = .08).Attitudes and interaction rates decreased between first and second follow-up (*t*(80) = 3.9, *p* = .0005). Increased job stress (*r* = .31, *p* = .002) and burnout (*r* = -.77, *p* = .001) were negatively associated with attitudes to PD. Better attitude to PD was associated with strong job performance (*r* = .31, *p* = .002).Greater enjoyment (APDQ scale) was associated with lower interaction rates with PDOs (*F*(1.77) = 9.17, *p* = .003, *η^2^* = .106).	High
32	Nathan et al. (2007)	28 Nursing staff at MSU: 14 working on a female ward 14 working on a male ward	Quantitative, Longitudinal: Survey design, Maslach Burnout Inventory to at baseline and 18 month follow up	4* (due to low statistical power)	Both groups experienced similarly low rates of expressed emotional exhaustion at baseline relative to normative data. Staff on the female ward showed higher rates relative to normative data at follow up (*d* = 1.70) *(NB*: *effect size appears inaccurate)*.Staff on the male ward showed a smaller increase at follow-up than those on female ward (*d* = .76).	High
21	Crichton & Calgie (2002)	Charge nurses responding to 31 incidents of inter-personal violence	Mixed: Semi-structured questionnaire	5	PD diagnosis was associated with blameworthiness and sanctions but not associated with other moral censure responses. Moral judgements about a patient’s blameworthiness influences staff responses.	Medium–High
20	Mason et al. (2009)	78 forensic nurses in low and medium secure settings	Quantitative, C/S: Survey of role construct definitions	5	Significant differences found between the constructs of PD management and PD clinical suggesting that nurses endorse management over intervention for PDOs.	Medium
22	Viukari et al. (1979)	36 staff members: 16 nurses, 20 Physicians	Quantitative, C/S: Rating of a 'sympathy scale' for 12 disorders	5	For both groups 'psychiatric assessment of criminals' elicited the least sympathy.	Medium
39	MacPhail & Beck-Sander (1999)	61 untoward incidents at an MSU in a 6 month period committed by 36 patients	Quantitative, Panel design: Analysis of serious incidents.	4	Patients detained under Psychopathic Disorder perpetrated a higher proportion of incidents (57%) than those detained under MI (43%) despite fewer PD patients (n = 13) than MI (n = 23).Female PD patients over-represented in incidents (56%) (represented 17% of the sample).	Medium
25	Graham (1980)	100 outpatient therapists	Quantitative, Case control: 49 allocated offender case files and 51 allocated non-offenders, followed by questionnaire.	4	PDOs regarded as least appropriate for therapy, least likely to be selected, least motivated, least likely to make progress, and most likely to drop out, but not different in capacity for insight. Non-offenders were rated more appropriate for therapy. Offenders were not found to attract a significantly higher rate of PD diagnosis.	Low
23	Bowers et al. (2005)	73 Prison officers from UK Dangerous and Severe Personality Disorder (DSPD) Prison Unit; 59 at 8 month follow up 37 at 16 month follow up	Mixed, Longitudinal: Thematic analysis of semi-structured interviews	5	Numbers of positive thematic reports (n = 527) and negative reports (n = 521) were comparable. 'Positive' themes: seeing prisoners as individuals; understanding behavioural patterns on which change could be facilitated; and developing a positive therapeutic relationship. ‘Negative' themes: negative portrayal in the media; behaviours of manipulation, self-injury and attention-seeking promoted feelings of intolerance, frustration, disinterest in prisoners, and staff feeling de-skilled, under-confident and stressed.	High
28	Fortune et al. (2010)	22 staff multi-disciplinary staff from 3 Medium Secure Units (MSUs)	Qualitative: Thematic analysis applied to semi-structured interviews	5	Clinical work was ‘relentless’ and ‘draining’; daily work environment was stressful. Almost all staff felt afraid of service users at some point. Staff underestimated the emotional impact of clinical work, in particular those engaged in regular face-to-face contact.	High
30	Tetley et al. (2011)	20 staff members from medium and high secure units	Qualitative, C/S: thematic analysis of semi-structured interviews	5	Challenging & inappropriate behaviour by PDOs (complaining, pushing boundaries, verbal/physical aggression).Motivational and engagement problems with patients.Limited communication between services.	High
40	Kurtz & Turner (2007)	13 staff from a forensic MSU PD Unit	Qualitative, C/S: Semi-structured interviews analysed using grounded theory method	5	Negative findings include: i) staff feel both physically and psychologically cut off from society and from other staff groups within the same establishment who did not work with PDOs; ii) feelings of frustration when PDOs are not open regarding their offences; iii) a struggle to connect the victimized and victimizing aspects of patients, integrating aggression with vulnerabilities; iv) senior practitioners were somewhat drained, overburdened and burned out. Positive findings: i) clinical work with PDOs is both difficult and different, but also exciting and cutting edge; ii) there is a sense of purpose related to work; iii) challenges of the work and developing a real understanding of offender’s problems are a source of satisfaction; iv) staff feeling physically safe and no stress regarding personal safety or public protection responsibilities.	High
41	Kurtz & Jeffcote [37]	25 MSU staff: 12 staff from a PD unit and13 from a mainstream unit	Qualitative, C/S: Semi-structured interviews analysed using grounded theory method	5	Staff difficulties include: i) reconciling PDOs’ offending behaviour and level of violence with their presentation; ii) viewing offenders as vulnerable as this contradicts with their knowledge of offenders’ potential to abuse others; iii) Regarding therapeutic and custodial tasks as distinct. Positive aspects for staff include: i) forensic wards are physically safe places, with minimal sense of risk and anxiety; ii) a lack of stress in direct work with patients compared to what outsiders would imagine; iii) new staff are at greater risk from patients than established staff.	High
44	Turley et al. (2013)	Approximately 24 staff from 3 PIPES sites	Qualitative, C/S: thematic analysis of interviews and mini-group discussions	5	Staff had more positive attitude towards PDOs because of their involvement in PIPES Group supervision gave staff more skills in interacting with PDOS and enabled them to develop a deeper understanding of PDOs’ behaviour	High
17	Boyle et al. (2009)	5 staff members	Qualitative, Cross-Sectional (C/S): 5 semi-structured interviews applying a modified grounded theory approach	5	‘PD’ labels possess negative connotations and staff react in ways that were not always therapeutic as a result of labels. Reactions, counter-transferences, thoughts or emotions caused by PDOs can lead to harmful consequences and psychological injury for patients.	Medium
24	Grounds et al. (2004)	55 clinicians responsible for admission to MSU	Qualitative, C/S: Semi-structured interviews	5	47% clinicians reported patients with a primary diagnosis of severe PD considered unsuitable for medium security because they were considered untreatable, could block beds, and/or frequently caused disruption among staff and patients.	Medium
27	Department of Health and Ministry of Justice National Offender Management Service (2011)	N/A	Expert opinion / review	5	Reactions to PDOs include: feeling puzzled and irritated; frustration; helpless to help them change; defensive; fearful of upsetting the person and getting into an argument and manipulated. Staff feel exhausted, burnt out, personalise their responses, and feel critical towards PDOs and lose capacity for empathy which leads staff to become punitive and hostile, over-involved, and avoid PDOs.Dysfunctional personality traits can emerge in staff so that unexpected outbursts of extreme hostility or rigidity occur, or entangled or overly involved alliances with PDOs commence.	High
29	Moore & Freestone (2006)	N/A	Expert opinion	5	Work with high-risk PDOs can lead to ‘factioning’ or splitting in staff teams. The process of creating holding environments for these individuals can be expected to go through ‘stormy’ periods of violence and acting out.	High
31	McMillan (2004)	N/A	Expert opinion	5	Female PDOs in high secure settings can be emotionally exhausting and intense.	High
36	Clarke & Ndegwa (2006)	N/A	Expert opinion	5	Staff can forget about pathology and find it difficult to control their counter-transference reactions to PDOs. Managers of programmes for PDOs must be clinically informed.	High
38	Morris (2003)	N/A	Expert opinion	5	The relationship between staff and PDOs is the “arena of pathology” (p. 79). PDOs find ways of undermining and 'getting around’ treatment. Staff are likely to be subject to unexpected enactments, dynamics and manipulations.	High
13	Kurtz (2005)	N/A	Expert opinion	5	Staff are satisfied with their work with PDOs but also experience stress, and nursing staff are likely to develop burnout. A pessimistic view of treatment efficacy can be reduced if staff are made aware of evidence base for interventions. Nurses’ contact with patients is not viewed by staff as producing more stress than organisational factors, but feelings of anxiety and frustration related to relationships with PDOs may be transferred onto issues that are external and concrete.	Medium–High
33	Crichton (1998)	N/A	Expert opinion	5	From a psychodynamic perspective, violent/threatening/disruptive PDOs may engender hate in the countertransference. Changes in nursing care erode traditional mechanisms of institutional defence and may contribute to an increase in staff anxiety.	Medium
34	Evans (2011)	N/A	Expert opinion	5	Without clinical supervision staff may react to ASPD patients’ projections by pushing them back into the patient in an aggressive or premature way to protect their own sanity or sense of professionalism; re-enacting a sadistic counter-transference. Staff can have strong reactions to PDOs leaving them feeling helpless, ineffective, intimidated, frightened or ‘pinned against the ropes’ where they either act out in response to patients’ provocations or distance themselves for fear of acting out.	Medium
35	Ruszcynski (2010)	N/A	Expert opinion paper	5	Violent PDOs may cause feelings of fright or of being violated (staff may react sadistically).Working with sexually perverse patients may cause staff to feel: disgusted/corrupted (sometimes voyeuristic or seduced) and/or defensively sadistic and abusive, and may engage in minimising and denial of behaviours leading to destructive staff dynamics.	Medium
37	Protter & Travin (1983)	N/A	Expert opinion paper	5	Without clinical supervision and support groups, unaddressed counter-transference responses to patients cause anger/rage, helplessness/hopelessness, denial, boredom, over-responsibility and despair.	Medium

Four of the 10 identified experts responded to a request for information and suggested a further 7 potential, unpublished, papers. No papers identified by either route were deemed relevant to the study objectives: the primary reasons being either: a lack of outcome, due to the study being unfinished at that point; or having an outcome relating to patients, rather than staff.

Hand-searching of the top five journals (listed in S1 Appendix) produced a further 14 possible papers. No papers identified were relevant to the study on full-text review.

#### Areas of impact identified

Following review of the articles identified by the initial searches, six areas of impact emerged as relatively discrete themes and were used subsequently to better structure and synthesise data effectively. These were:

*Attitudes and experience* (13 papers)

*Burnout* (5 papers)

*Counter-transference* (or psychodynamic impact more broadly defined) (5 papers)

*Perceived risk of violence* (2 papers)

*Job satisfaction* **(2 papers)**

*Stress* (3 papers)

Stress as an outcome featured in three papers, but was not utilised as a primary outcome in any paper.

Depending on their primary theme or outcome variable(s), studies were classified into one or more of these areas of impact. Where studies fell into more than one identified area of impact, they were classified according to their primary outcome.

#### Methodological quality of included studies

Of the identified papers, ten were expert opinion papers and did not follow a scientific methodology. Of the remaining papers, nine employed a quantitative methodology, typically a cross-sectional survey design (5 papers); also including cost-benefit analysis (2 papers); or simple statistical description of clinical records (2 papers). A further eight studies utilised a qualitative methodology.

The overall quality of the evidence identified by the search was very low according to the hierarchy proposed by Greenhalgh [17] and operationalised by the CEBM checklist [16]. No previous systematic reviews or meta-analyses were identified, and no studies featured RCTs or quasi-experimental methods. This was not unexpected given that the review was not of an intervention, but related to exposure. Only one identified study [18]met the criteria for Level 3 Evidence according to the CEBM checklist as an adequately statistically powered, non-randomised follow-up study. Four further studies [19, 20] employed a form of non-randomised longitudinal design: however, one was qualitative [19]; two more were case-control only [21, 22]; and the fourth [20] was inadequately powered to detect a common harm due to working with PD offenders, and was therefore downgraded from Level 3 to Level 4 Evidence.

The remaining identified papers (n = 23; 85%) involved either descriptive (cross-sectional or qualitative) methodologies, or were simple ‘case studies’ of organisations and were classified as the lowest level of evidence (Level 5). Qualitative studies tended to focus on a single discipline—usually 'frontline' staff such as nurses or prison officers—and did not take multidisciplinary working into account. Quantitative studies identified suffered from a number of general weaknesses, including: low sample sizes; heterogeneity of outcomes; lack of a clear assessment of PD; no control for confounding variables.

### Narrative summary of results

#### Attitudes and experience

This area of impact contained the highest proportion of quantitative papers (five, 42%), including cohort and case-control studies, and the lowest proportion of ‘expert opinion’ pieces (only one, 8.3%), suggesting that the evidence base for attitudes of staff to working with PDOs may be more developed than in other impact areas.

Overall, work with PDOs inspired negative attitudes amongst staff. Across criminal justice and hospital settings, the label of ‘personality disorder’ was associated with negative connotations, and staff reacted in a less therapeutic or more ‘managerial’ way to individuals with this label [23–26]. Staff felt that PDOs inspired a greater sense of blameworthiness and susceptibility to censure [27] and lower levels of sympathy [28]. Attitudes to working with PDOs was found to show a trend toward becoming more negative with increased duration of exposure [19].

Nurses considered PDOs difficult to treat and to engage in treatment. They lacked confidence in the efficacy of clinical interventions [13, 24, 25, 29] and believed that PDOs were least likely to make progress and most likely to drop out of treatment, relative to other patients [21].

A guide document produced by the UK Ministry of Justice [30] suggested that PDOs evoked reactions in staff including: puzzlement and irritation; frustration; helplessness; defensiveness; fear and feelings of being manipulated, causing staff to lose the capacity for empathy and become more punitive towards PDOs.

Staff with a sense of enjoyment of their job and strong job performance showed a more positive attitude to their work with PDOs [18]. Staff involved in working in Psychologically Informed Planned Environments (PIPES) reported a more positive attitude towards PDOs [31].

#### Burnout

The quality of evidence in this area of impact was relatively good with only one identified paper being expert opinion. In her narrative review, Kurtz highlighted that holding negative attitudes to PDOs was associated with job stress, burnout and possible vicarious traumatisation [13]. Work with female PDOs in high security was reported to be emotionally exhausting and intense for nurses in particular [32]. An increased emotional burden associated with working with female, when compared with male, PDOs at follow-up was also described by Nathan et al. [20]. Bowers et al. [19] found that a sense of frustration caused through working with PDOs caused prison staff to feel de-skilled, under-confident and stressed.

Challenging and inappropriate behaviour by PDOs was also thought to be draining, stressful and to inspire a degree of fear [33]. Such behaviour could also lead to splits within the staff team itself [34] or difficulties with communication [35] that could deepen over time.

Contact with PDOs was not viewed as producing as much stress as ‘organisational factors’ [13]. Kurtz and Turner [36] found that staff working with PDOs reported feeling physically and psychological ‘cut off’ from both society and professionals working with other kinds of patients. The lack of openness of PDOs was also noted as particularly frustrating. They noted that senior practitioners felt particularly drained, overburdened and burned out. In a later study relying on a mixed sample including some mentally ill offenders, Kurtz and Jeffcote [37] confirmed these findings, including the confusion often elicited in staff members by work with PDOs, in attempting to reconcile the patients’ vulnerabilities with their potential to abuse of others; and the preoccupation that can arise with staff dynamics in this group.

#### Counter-transference

The quality of evidence in this area of impact was low given that it was based entirely on expert opinion papers.

A number of papers written from a psychodynamic perspective suggested that work with PDOs was associated with negative counter-transferential experiences and hate in the countertransference [38] amongst staff. This could be sadistic [39, 40] and hard to control [41], often resulting in fear, defensiveness, anger, rage, helplessness/ hopelessness, denial, boredom, over-responsibility and despair [42]. One paper hypothesised that the implication of these countertransference reactions, particularly defensiveness, had a destructive effect on overall organisational dynamics that could be ‘effectively invisible’ [40]. Morris [43] observed that the high level of competence of PDOs to attack and circumvent treatment efforts subjected staff to unexpected negative dynamics.

#### Perceived risk of violence

The quality of evidence in this area of impact was relatively high given that it was based on data-based research and not expert opinion papers.

One study found that patients identified as having Psychopathic Disorder were responsible for a higher proportion of violent and property-destructive incidents than patients with Mental Illness [22]; however, a second study by Crichton and Calgie [27] did not identify a higher risk of violence posed by PDOs but did suggest that this patient group were seen as more ‘blameworthy’.

Two studies [36, 37] identified a minimal sense of risk and anxiety associated with work in forensic settings ***per se***, and also noted that greater experience in working with PDOs was associated with a perception of decreased risk to staff.

#### Job satisfaction

The quality of evidence in this area of impact was relatively good with only one identified paper being expert opinion.

**J**ob enjoyment was associated with lower rates of staff interaction with PDOs [18]. Clinical work with PDOs was described as ‘exciting and cutting edge’ [36]; this was confirmed by Kurtz in her narrative review, where she concluded that people who work with PDOs tended to be satisfied with their work [13]. In particular, the challenges of the work and developing a real understanding of patients’ problems were sources of satisfaction [36].

## Discussion

Our review confirmed that the evidence base is sparse, heterogeneous and used methodologies generally considered to be of a low level according to standard classifications [17]. The lack of use of standardised assessment of factors related to impact areas limited the robustness and generalizability of findings. However, the evidence identified suggests that working with PDOs is associated with negative attitudes, burnout, stress, and negative counter-transferential experiences, whilst perceived risk of violence related to PDOs is experienced according to the amount of experience working with PDOs, such that those with more experience perceive less of a risk. Despite the predominance of negative impact areas, positive experiences of excitement from being involved in innovative services for PDOs were identified.

### Overall completeness and applicability of evidence

Although many studies identified the need for interventions to improve the health and wellbeing of staff working with PDOs, no studies were identified that evaluated a specific intervention, even with a quasi-experimental methodology. This lack of evaluation of interventions limits the generalisability and applicability of the evidence identified to forensic services; however, the findings of several studies relating to the specific negative effects of working with PDOs (namely: hardening of attitudes; staff burnout; diminished job satisfaction; negative countertransference; exposure to violence; and job stress) may provide a basis for the future identification of interventions directed at improving the staff experience.

### Potential biases in the overview process

The studies identified by this review showed little control for bias. Even after excluding the clinical papers, which did not follow any form of scientific or experimental design, there were a number of biases in the studies employing a clear methodology.

Selection bias: studies employing survey methodologies did not allow for possible systematic differences between self-selected responders and non-responders in terms of the variables under investigation. Response rates for cross-sectional survey studies tended to be relatively low, ranging from 35–55%.

Population bias: most studies tended to focus on a single professional discipline, typically or prison officers. The few studies that included a range of professions were often qualitative in nature. One quantitative study did include multiple professional groups outside of nursing but considered these as a homogeneous group when compared with nurses.

Measurement bias: there seemed to be little agreement about appropriate measurements of impact on staff, and studies utilised a range of outcome measures, ranging from change in attitudes to burnout and violent incidents. Only one study adopted a longitudinal methodology.

Exposure bias: although all studies considered offender groups, some studies were conducted in treatment settings where the patient group was defined by obsolete categories such as the UK medico-legal category of ‘Psychopathic disorder’. Such categories will have included some patients with disorders other than PD. One study was conducted with a homogeneous group of PDOs and mentally ill offenders, although these were separated to an extent in the analysis.

## Conclusion

Although not the focus of this review, this study identified a lack evidence for interventions intended to moderate the impact of working with PDOs. Whilst Turley et al.[31] noted that involvement in group supervision helped equip staff with skills in interacting with PDOs and improved their ability to understand the behavioural motives of PDOs, many interventions (staff training; staff support; counselling; clinical supervision etc.) were frequently alluded to in the literature but not subsequently evaluated.

Services for the assessment and management of PDOs have expanded considerably over recent years. However, the evidence for their effectiveness, and cost- effectiveness, has thus far been equivocal [44]. Randomised controlled trials or robust quasi-experimental studies of interventions aimed at moderating the negative impact of working with PDOs on staff are now important in order not only to better meet the needs of this challenging clinical population but also encourage the development of a sustainable workforce and to optimise the clinical and risk outcomes of services.

### Excluded Studies

A list of excluded studies is obtainable from the authors at request.

## Supporting Information

S1 Appendix List of Hand-Searched Journals. (DOCX)

S2 Appendix Data Extraction Forms. (DOCX)
