# Supplementary material for: The Impact on Staff of Working with Personality Disordered Offenders: A Systematic Review
Source: PLoS One. 2015 Aug 25;10(8):e0136378. doi: 10.1371/journal.pone.0136378 (PMC4549262; doi:10.1371/journal.pone.0136378)
Supplement: S1 Appendix — (DOCX) [file pone.0136378.s001.docx]

# S1 Appendix: List of Top five journals for hand-searching

- 1. Journal of Forensic Psychiatry and Psychology
  2. Criminal Behaviour and Mental Health
  3. The Journal of Psychiatric and Mental Health Nursing
  4. International Journal of Therapeutic Communities
  5. British Journal of Forensic Practice
