# Supplementary material for: The Impact on Staff of Working with Personality Disordered Offenders: A Systematic Review
Source: PLoS One. 2015 Aug 25;10(8):e0136378. doi: 10.1371/journal.pone.0136378 (PMC4549262; doi:10.1371/journal.pone.0136378)
Supplement: S2 Appendix — (DOCX) [file pone.0136378.s002.docx]

# S2 Appendix: Data Extraction Forms

Data extraction table for expert opinion papers

| Title of review |  |
| --- | --- |
| Author/s |  |
| Year of publication |  |
| Publication type |  |
| Title of publication (volume, issues, page numbers) |  |
| Background/literature review |  |
| Aim/s as stated by author |  |
| Type of profession/setting considered |  |
| Constructs/topics of interest |  |
| Authors’ conclusions |  |
| Comments |  |

Data extraction table for single qualitative papers

| Title of study |  |
| --- | --- |
| Author/s |  |
| Year of publication |  |
| Publication type |  |
| Title of publication (volume, issues, page numbers) |  |
| Setting/s (HS DSPD, MS DSPD, forensic MI, general adult inpatient, etc.) |  |
| Degree of relevance of setting to MS PD pilots  High, medium, low relevance |  |
| Literature review (main/important points) |  |
| Aim/s of study as stated by authors |  |
| Which of our Q-s does this study address:  1. Impact |  |
| 2. Factors related to impact |  |
| 3. Strengths of relationships |  |
| 4. Model describing predictors, moderators, mediators, outcomes, etc. |  |
| 5. Factors identified as relevant to staff selection, training, support, and supervision. |  |
| 6. Other, specify |  |
| Professional group/s |  |
| Number of participants |  |
| Response rate |  |
| Socio-demographic characteristics of participants (gender, age, ethnicity, class, marital status etc.) |  |
| Qualitative method used (ethnography, grounded theory, thematic analysis, content analysis, focus groups, life histories, etc.) |  |
| Stressor/exposure |  |
| Outcomes |  |
| Model specified |  |
| Results |  |
| Quality appraisal (relevance, clarity of research question, appropriateness of design to question, context, sampling, data collection and analysis [audit trail], reflexivity, triangulation, respondent validation, attention to negative cases, fair dealing, etc.) |  |
| Authors conclusions |  |
| Comments |  |

Data extraction table for single quantitative studies

| Title of study |  |
| --- | --- |
| Author/s |  |
| Year of publication |  |
| Publication type |  |
| Title of publication (volume, issues, page numbers) |  |
| Setting/s (HS DSPD, MS DSPD, forensic MI, general adult inpatient, etc.) |  |
| Degree of relevance of setting to MS PD pilots  High, medium, low relevance |  |
| Background/literature review |  |
| Aim/s of study as stated by authors |  |
| Which of our Q-s does this study address:  1. Impact (with or without comparison group) |  |
| 2. Factors related to impact |  |
| 3. Strengths of relationships |  |
| 4. Model describing predictors, moderators, mediators, outcomes, etc |  |
| 5. Factors identified relevant to staff selection, training, support, and supervision. |  |
| Professional group/s (experimental group) |  |
| Professional group/s (comparison group) |  |
| Size of achieved samples |  |
| Response rate |  |
| Socio-demographic characteristics of experimental and control group  groups (gender, age, ethnicity, class, martial status etc) |  |
| Study design |  |
| Statistical analysis (e.g. hierarchical regression, ANOVA, etc) |  |
|  | |
| Stressor/exposure |  |
| Factors outside job |  |
| Organizational factors |  |
| Job-related factors |  |
| Patient-related |  |
| Personal characteristics |  |
| Mediators |  |
| Moderators |  |
| Outcomes (measure of impact)  (e.g. stress, burnout, job satisfaction, feelings, etc.) |  |
| Model specified |  |
| Results (effect size if possible)   1. Impact 2. Factors related to impact 3. Strengths of relationships 4. Model 5. Factors relevant to staff selection, training, support, and supervision |  |
| Study quality:   1. Construct V of Ms used |  |
| 1. Statistical conclusion V (incl. power) |  |
| 1. Internal V |  |
| 1. External V |  |
| 1. Descriptive V |  |
| Authors conclusions |  |
| Comments |  |
